# On the brain structure heterogeneity of autism: Parsing out acquisition site effects with significance‐weighted principal component analysis

**DOI:** 10.1002/hbm.23449

**Published:** 2016-10-24

**Authors:** Francisco Jesús Martinez‐Murcia, Meng‐Chuan Lai, Juan Manuel Górriz, Javier Ramírez, Adam M. H. Young, Sean C. L. Deoni, Christine Ecker, Michael V. Lombardo, MRC AIMS Consortium,, Simon Baron‐Cohen, Declan G. M. Murphy, Edward T. Bullmore, John Suckling

**Affiliations:** ^1^ Department of Signal Theory Networking and Communications, C/Periodista Daniel Saucedo Aranda S/N E‐18071, University of Granada Granada Spain; ^2^ Child and Youth Mental Health Collaborative at The Centre for Addiction and Mental Health and The Hospital for Sick Children Toronto Ontario Canada; ^3^ Department of Psychiatry University of Toronto Toronto Ontario Canada; ^4^ Department of Psychiatry, Autism Research Centre University of Cambridge Cambridge United Kingdom; ^5^ Department of Psychiatry National Taiwan University Hospital and College of Medicine Taipei Taiwan; ^6^ Advanced Baby Imaging Laboratory, School of Engineering Brown University Providence Rhode Island; ^7^ Department of Forensic and Neurodevelopmental Sciences, Sackler Institute for Translational Neurodevelopment London United Kingdom; ^8^ Institute of Psychiatry, Psychology and Neuroscience, King's College London London United Kingdom; ^9^ Department of Psychology and Center for Applied Neuroscience University of Cyprus Nicosia Cyprus; ^10^ Cambridgeshire and Peterborough NHS Foundation Trust Cambridge United Kingdom; ^11^ Department of Psychiatry, Brain Mapping Unit University of Cambridge Cambridge United Kingdom

**Keywords:** autism spectrum disorder, structural magnetic resonance imaging, structural heterogeneity, voxel based morphometry

## Abstract

Neuroimaging studies have reported structural and physiological differences that could help understand the causes and development of Autism Spectrum Disorder (ASD). Many of them rely on multisite designs, with the recruitment of larger samples increasing statistical power. However, recent large‐scale studies have put some findings into question, considering the results to be strongly dependent on the database used, and demonstrating the substantial heterogeneity within this clinically defined category. One major source of variance may be the acquisition of the data in multiple centres. In this work we analysed the differences found in the multisite, multi‐modal neuroimaging database from the UK Medical Research Council Autism Imaging Multicentre Study (MRC AIMS) in terms of both diagnosis and acquisition sites. Since the dissimilarities between sites were higher than between diagnostic groups, we developed a technique called Significance Weighted Principal Component Analysis (SWPCA) to reduce the undesired intensity variance due to acquisition site and to increase the statistical power in detecting group differences. After eliminating site‐related variance, statistically significant group differences were found, including Broca's area and the temporo‐parietal junction. However, discriminative power was not sufficient to classify diagnostic groups, yielding accuracies results close to random. Our work supports recent claims that ASD is a highly heterogeneous condition that is difficult to globally characterize by neuroimaging, and therefore different (and more homogenous) subgroups should be defined to obtain a deeper understanding of ASD. *Hum Brain Mapp 38:1208–1223, 2017*. © 2016 Wiley Periodicals, Inc.

**Abbreviations**
AAL: Advanced Automated Labelling
AAM: Active Appearance Models
ABIDE: Autism Brain Imaging Data Exchange
ADNI: Alzheimer's Disease Neuroimaging Initiative
ADOS: Autism Diagnostic Observation Schedule
ANOVA: Analysis of variance
ASD: Autism Spectrum Disorder
CBM: Component‐based morphometry
CSF: Cerebrospinal fluid
GM: Grey matter
IR: Inversion Recovery
MNI: Montreal Neurological Institute
MRI: Magnetic resonance imaging
PCA: Principal component analysis
SBM: Source based morphometry
SPGR: Spoilt gradient recall
SSPF: Steady state free procession
SVC: Support vector classifier
SVD: Singular value decomposition
SWPCA: Significance‐weighted principal component analysis
VBM: Voxel‐based‐morphometry
WASI: Wechsler abbreviated scale of intelligence
WM: White matter

## INTRODUCTION

Autism Spectrum Disorder (ASD) is a neurodevelopmental syndrome characterized by social and communication impairment as well as restricted, repetitive patterns of behaviour, interests or activities. The delimitation of both functionally and structurally affected areas in the brain in such an etiologically and neurobiologically heterogeneous condition has long been a major concern [Ecker and Murphy, 2014; Lai et al., 2013; Lenroot and Yeung, 2013]. With this context, the use of large samples is of fundamental importance, and has been addressed by establishing multi‐centre collaborations such as the UK Medical Research Council Autism Imaging Multi‐centre Study (MRC AIMS) [Ecker et al., 2012, 2013, 2015] and the Autism Brain Imaging Data Exchange (ABIDE) [Di Martino et al., 2014]

Multicentre studies with structural (sMRI) and functional magnetic resonance imaging (fMRI) are increasingly common, allowing for recruitment of larger samples in shorter periods of time. However, the use of images acquired at different sites still poses a major challenge. In addition to logistical difficulties, such as regulatory approvals and data protection, a number of technical and methodological issues can potentially affect the resulting maps, introducing undesired intensity and geometric variance. This issue has been addressed in other neurological conditions, such as Alzheimer's Disease [Jovicich et al., 2006; Stonnington et al., 2008], where group differences are well known, and demonstrating that the impact of a correction for site on the resulting neurobiological differences is relatively small. However, these effects have a stronger impact in psychiatric conditions where the atypical radiological signs on MRI are often subtle and require large samples of patients to observe on‐average differences relative to control samples. Recent meta‐analyses point to differences being inconsistently reported in schizophrenia [Friedman and Glover, 2006; Turner et al., 2013], psychosis [Clementz et al., 2016; Wang et al., 2015], and ASD (using the multi‐centre ABIDE database) [Haar et al., 2014].

These inconsistencies can arise from a variety of variance sources, ranging from the multi‐level (phenotypic, neurobiological, and etiological) heterogeneities of the conditions to technical issues that include differences in scanner make, model, manufacturer, static field strength, field inhomogeneities, slew rates and image reconstruction [Van Horn and Toga, 2009], as well as acquisition problems such as within‐acquisition participant head motion. Field inhomogeneities are a source of misinterpretation of the data even when the same MRI system manufacturer and model are used [Van Horn and Toga, 2009]. Furthermore, results in [Pearlson, 2009] demonstrate that a single scanner can change with time, which makes some widely used strategies, for example collecting controls first and patients later, a flawed approach. Recent neuroimaging research on ASD [Haar et al., 2014] has shown that, while analyses performed on a particular database (acquired on a single platform) could yield coherent regions, the atypical structures are often inconsistent across the wider literature using different databases. Therefore, new methodologies focused on reducing multi‐site variance may be potentially helpful in increasing the power to identify the characteristic neurobiological signature of autism, should there be one.

A number of approaches have been proposed to reduce between‐site variance. Geometric distortions caused by magnetic fields inhomogeneities have been widely studied [Jovicich et al., 2006; Stonnington et al., 2008]. Furthermore, there exist a number of diffeomorphic registration algorithms, such as DARTEL [Ashburner, 2007] or ANTS [Avants et al., 2010], intended to reduce inter‐subject—and by extension, inter‐site—variations. In the case of intensity variance, the issue has already been addressed in the MRC AIMS database by leveraging sMRI sequences that yield quantitative estimates of relaxation times [Deoni et al., 2008] that have been demonstrated to reduce single‐site effects compared to weighted sequences. However, images acquired using this technique still yield between‐site differences [Suckling et al., 2014].

To address the problem of intensity variance and improve the homogeneity of the images across different sites, we have developed a new post‐acquisition methodology that enhances derived maps (e.g. grey or white matter volumes) by means of ameliorating site effects. This method, called significance‐weighted principal component analysis (SWPCA), can be applied as part of pre‐processing before computing whole‐brain or regional statistical analysis. The algorithm proceeds by performing a principal component analysis (PCA) over the whole database of images and later computing the statistical significance of each component in relation to a categorical variable, in this case the acquisition site. This information is used to reconstruct the datasets using a weighted strategy that effectively reduces intensity inhomogeneities due to site effects.

This article is organised as follows: First, in “Material and Methods” section, the methodology is presented. It comprises the presentation of the MRC AIMS image database and its pre‐processing, PCA, one‐way analysis of variance (ANOVA), and how these two statistical procedures are combined in a weighted approach to create the corrected maps. In “Results” section, the algorithm is applied to maps of grey and white matters and estimates of relaxation times, with qualitative and quantitative results presented before and after applying SWPCA to the images. “Discussion” section is used to discuss the relevance of the SWPCA algorithm and the results of the case‐control comparison, and finally in “Conclusions” section, we draw conclusions concerning multi‐site studies and the neurobiology of autism.

## MATERIAL AND METHODS

### Image Database

Structural MRI were analysed from 136 adult, right‐handed males (68 with ASD and 68 matched controls) with no significant mean differences in age and full‐scale IQ, acquired from the centres contributing to the UK Medical Research Council Autism Imaging Multi‐centre Study (MRC AIMS) [Ecker et al., 2012, 2013, 2015] and recruited by advertisement. In this work, only participants recruited at the Institute of Psychiatry, King's College London (LON) and the Autism Research Centre, University of Cambridge (CAM) were included where an equivalent set of images were acquired from each participant.

Participants were excluded from the study if they had a history of major psychiatric disorder or medical illness affecting brain function (e.g. psychosis or epilepsy), or current drug misuse (including alcohol), or were taking antipsychotic medication, mood stabilizers or benzodiazepines.

All participants with ASD were diagnosed according to International Classification of Diseases, 10th Revision (ICD‐10) research criteria, and confirmed using the Autism Diagnostic Interview‐Revised (ADI‐R) [Lord et al., 1994]. Autism Diagnostic Observation Schedule (ADOS) [Lord et al., 2000] was performed, but the score was not considered as an inclusion criteria. ASD participants, to be included, must have scored above the ADI‐R cut‐off in the three domains of impaired reciprocal social interaction, communication and repetitive behaviours and stereotyped patterns, although failure to reach cut‐off in one of the domains by one point was permitted. Intellectual ability was assessed using the Wechsler Abbreviated Scale of Intelligence (WASI) [Wechsler, 1999], ensuring the participants fell within the high‐functioning range on the spectrum defined by a full‐scale IQ > 70. The demographics of the participants are shown in detail in Table 1.

**Table 1:** Demographics of the participants included in the analysis

Database	Group	*N*	Age (*μ* ± *σ* years)	IQ (*μ* ± *σ*)
LON	ASD	39	28.74 ± 6.52	111.28 ± 13.13
	CTL	40	25.30 ± 6.62	104.67 ± 11.16
CAM	ASD	29	26.83 ± 4.64	115.83 ± 11.88
	CTL	28	26.75 ± 7.32	115.25 ± 13.67
ALL	ASD	68	25.90 ± 6.95	109.03 ± 13.31
	CTL	68	27.93 ± 5.87	113.22 ± 12.81

Structural MRI were obtained using Driven Equilibrium Single Pulse Observation of T1 and T2 (DESPOT1, DESPOT2) [Deoni et al., 2008] at King's College London and University of Cambridge, both with 3T GE Medical Systems HDx scanners. Using multiple spoilt gradient recall (SPGR) acquisitions in the DESPOT1 sequence and steady state free procession (SSPF) acquisitions in the DESPOT2 sequence, with different flip angles and repetition times, quantitative T1 and T2 maps (qT1 and qT2) were calculated with a custom ImageJ plug‐in package. Correction of main and transmit magnetic field (B0 and B1) inhomogeneity effects was performed during the estimation of T1 and T2.

For accurate registration to the standard stereotatic space of the Montreal Neurological Institute (MNI), a simulated T1‐weighted Inversion Recovery (IR) images (synT1) were created based on the qT1 maps (Ecker et al., 2012, 2013; Lai et al., 2012]. The synT1 images were then segmented using New Segment into grey (GM) and white matter (WM) maps, and normalized to the MNI space using DARTEL in SPM8 [Friston et al., 2007], with modulation (preserve volume) to retain information of regional/local GM and WM volumes, and smoothed with a 3mm FWHM Gaussian Kernel to account for inter‐subject mis‐registration. The synT1, qT1 and qT2 maps were also registered to the standard MNI space using the same DARTEL flow fields, but without modulation (preserve concentration) to retain information of regional/local T1 contrast, T1 relaxation time, and T2 relaxation times, and smoothed with a 3mm FWHM Gaussian kernel. Therefore, there were five different modalities: qT1, qT2, synT1 map, GM and WM maps, for each every participant, which allows us to observe the impact of our SWPCA correction of site‐related undesired variance on quantitative (qT1 and qT2), simulated (synT1) images and probability maps (GM and WM).

During the pre‐processing of the images, several procedures targeted the reduction of inter‐subject and inter‐site geometric distortion, amongst them the correction of B0 and B1 field inhomogeneity effects and the registration to MNI space. Many other algorithms have been proposed to help in this task. However, the study of their relative performance lies beyond the scope of this article. Following image registration, it was assumed that only the intensity of the maps was affected between sites.

### Intracerebral Mask

Prior to any processing of the brain images, masks were applied that restricted the analysis to the brain parenchyma. These binary masks comprised voxels in the DARTEL study‐specific template with GM (or WM) probabilities >0.3, whilst excluding voxels with a cerebrospinal fluid (CSF) tissue probability >0.3, to avoid including CSF regions where T1 and T2 values are extremely high. Throughout this work three masks were used: selectively GM (excluding WM and CSF); selectively WM (excluding GM and CSF); both GM and WM (excluding CSF).

### Significance Weighted Principal Component Analysis

**Figure 1:**
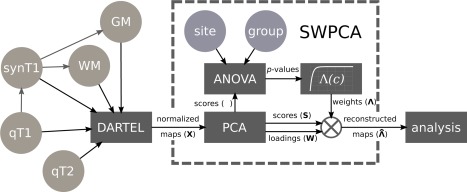
Summary of the SWPCA algorithm, along with its context in the pipeline used in this article. Circles represent the input data, both images (green shading) and class (group and acquisition site, purple shading). Rectangles represent the different procedures applied, comprising the DARTEL normalization and registration, the different steps contained in SWPCA ‐PCA, ANOVA and obtaining the weighting function Λ(c)‐ and the suseqent analysis.

The SWPCA is an algorithm to reduce, in this case, undesired intensity variance introduced by multi‐site image acquisition. SWPCA takes any dataset of pre‐processed images, spatially normalized, and decomposes them into their variance components to then provide a corrected dataset where these undesired variance components have been reduced. To do so, PCA was applied to each modality in turn to obtain the component scores and component loadings. Since PCA is a data‐driven approach, it was only used to decompose the source images, and after this procedure, a one‐way ANOVA estimated the relation between each variance component and a given categorical variable, in our case, the acquisition site. The between‐site variability in the variance component was then identified by its corresponding *P*‐value. Finally, these *P*‐values were transformed into a weighting matrix Λ that weighted the influence of each variance component in a final PCA reconstruction of the corrected maps. The procedure is summarized in Figure 1.

#### Principal component analysis

The first step in the SWPCA algorithm was to perform a PCA decomposition of the dataset into a set of orthogonal components that model the variance present in the images, in an analogous way to the paradigm described in Active Appearance Models (AAM) [Cootes et al., 2001].

PCA is a statistical procedure that uses an orthogonal transformation to convert a set of observations X of possibly correlated variables, where  X is a *K* × *N* matrix, with *K* participants (in this case, with one image per participant) and *N* the number of voxels, into a set of *N* linearly uncorrelated variables called Principal Components (PC, also known as component loadings or the mixing matrix) W of size *N* × *N* whose linear combination using a vector of component scores $s_{K}$ can perfectly recompose each image. The set of these component scores S (size *K* × *N*) was estimated as:

(1) S=XW

This transformation computes a sequence of PCs, maximally explaining the variability of the data while maintaining orthogonality between components. PCA was computed using singular value decomposition (SVD):

(2) $$X=U\Sigma V^{*}$$

where U is an *K* × *K* orthogonal matrix, Σ is an *K* × *N* diagonal matrix with non‐negative real numbers on the diagonal, and the *N* × *N* unitary matrix $V^{*}$ denotes the conjugate transpose of the *N* × *N* unitary matrix V. With this decomposition both the component scores and estimates of the set of components loadings W were obtained. In this work the truncated form of SVD was used such that only the first *C* components were considered, where most of the variability of the data was concentrated:

(3) $$S_{C}=U_{C}\Sigma_{C}={XW}_{C}$$

where $S_{C}$ is the set of component scores using the first *C* components (size *K* × *C*). To achieve reasonable performance with minimal information loss, it was assumed that the number of components was the same as the number of images, *C = K*. Thus, a partial reconstruction of the original signal could be undertaken:

(4) $$\hat{X}=S_{c}A_{c}$$

where $A_{C}$ is the pseudoinverse of the truncated matrix of component loadings $W_{C}$, and $\hat{X}$ is the reconstructed set of images.

#### One‐way analysis of variance

The estimated PCs effectively model the variability of the image dataset. The next step was to assess each PC as a source of inter‐site variance with ANOVA. ANOVA estimates the *F*‐statistic, defined as the ratio between the estimated variance within groups and the variance between groups:

(5) $$F=\;\frac{MS_{within}}{MS_{between}}=\frac{SS_{within}/(G-1)}{SS_{between}/(K-G)}=\frac{\sum_{i}n_{i}{\left({\bar{Y}}_{i}\;-\bar{Y}\right)}^{2}/\left(G\;-1\right)}{\sum_{ij}{\left(Y_{ij}\;-\bar{Y_{i}}\right)}^{2}/\left(K-G\right)}$$

Where $MS_{within}$ and $MS_{between}$ are the mean squares within‐ and between‐groups respectively, *G* is the number of separate groups (in our case, two), $\bar{Y}$ is the sample mean of a certain feature (in our case, the sample mean of all *K* values of a given component score), ${\bar{Y}}_{i}$ is the sample mean of the features belonging to group *i = 1…G*, $Y_{ij}$ is the *j*_th_ observation of a feature belonging to group *i* and $n_{i}$ is the number of participants in the $i_{th}$ group. The *F*‐distribution allows an easy computation of *P*‐values, given the number of groups and degrees of freedom. The *F*‐statistic and *P*‐values were computed independently for each component score and acquisition site, and then used in the SWPCA algorithm.

#### Weighting function

To obtain a set of corrected maps, a new signal matrix of all maps of the same modality, $\hat{X}$, was estimated with the influence of the PCs with variance related to acquisition site, assessed via the *P*‐values, reduced. To do so, Eq. (4) was modified to include a square matrix Λ (dimension C × C) whose diagonal contains a weight ${\;\lambda }_{c}$ for each component that depends on its *P*‐value; that is,

(6) $$\hat{X}=S\Lambda A$$

The computation of each ${\;\lambda }_{c}$, for each component, was performed using the Laplace distribution, modified so that the weights were on the interval [0, 1]:

(7) $${\;\lambda }_{c}\left(p_{c},p_{th}\right)=1-e^{\frac{\;-p_{c}}{p_{th}}}\;\forall p_{c}\;\in \left[0,1\right]$$

where $p_{c}$ is the statistical significance of the *c*‐th component with respect to the acquisition site and $p_{th}$ is the statistical threshold for significance; that is, $p_{th}$=0.05. This weighting ensured that most of the components of variance that are not related to the acquisition site are kept unchanged, while at the same time it strongly reduces the influence of components with *P*‐values less than the threshold.

**Figure 2:**
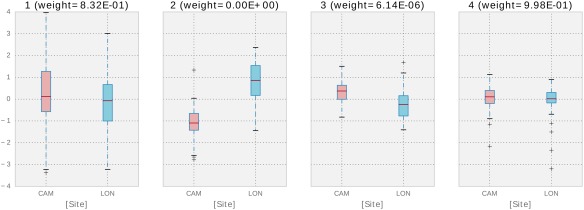
Box‐plot of the distribution of the component scores at each site in the four first components. We assume that bigger differences between distributions imply a bigger influence of the acquisition site on the portion of variance modelled by that component and therefore, to parse out those differences, the resulting weight will be smaller. [Color figure can be viewed at http://wileyonlinelibrary.com]

This procedure is illustrated in Figure 2, where a boxplot of the distribution of the first four principal component scores is shown. Since we have assumed that substantial differences imply a bigger influence of the acquisition site on the portion of variance modelled by that component, the resulting weight is reduced, and the contribution of that component to the reconstructed signal will be smaller. After computing all weights, most of the sources that are related to the acquisition site (for example, the second and third components) have been parsed out while keeping all other sources of variance.

### Experimental Settings and Validation

To validate the effects of the SWPCA algorithm on the inter‐site variance, experiments were undertaken to assess the reduction of the undesired site variance in the original datasets, and its impact on the between‐group signal. Two kind of analysis were performed: a characterization of voxel‐wise differences, and a classification analysis.

Voxel‐wise differences between groups were characterized using voxel‐based‐morphometry (VBM) [Ashburner and Friston, 2000], comprising preprocessing (registration, smoothing) and mass‐univariate *t*‐test on the smoothed maps from each modality. SWPCA is included (when needed) in this pipeline as a plug in, after the smoothing and before the computation of the test. Permutation testing assessed the significance of the relationship between the tested and target variables. A max‐type procedure was used to obtain family‐wise, whole‐brain corrected *P*‐values [Freedman and Lane, 1983]. Additionally, a component‐based morphometry (CBM), based on source based morphometry (SBM) [Xu et al., 2009] was used. This procedure provided *Z*‐maps for visual inspection comparable to those obtained in VBM, by selecting component loadings W, scaling them to unit standard deviation and weighting their contribution to the final map with their statistical significance, computed using the same permutation inference as in VBM.

A classification analysis was undertaken using a common classification pipeline [Khedher et al., 2015; López et al., 2009] consisting of preprocessing, feature extraction and classification. SWPCA is used as a plug‐in here as well, after the preprocessing and before the feature extraction step. We used PCA on the images for feature reduction and a Support Vector Classifier (SVC) with linear kernel, as implemented in LIBSVM [Chang and Lin, 2011], to classify the component scores in both corrected and uncorrected datasets (i.e. with and without SWPCA).

The classification was validated using stratified 10‐fold cross‐validation [Kohavi, 1995]. In brief, 9 subsets of the dataset were used for extraction of the PCs and training of the classifier with the remaining subset used for testing. This procedure was repeated for each subset, repeated 10 times to avoid possible bias and random effects of the partitions. The average and standard deviation of the accuracy (acc), sensitivity (sens) and specificity (spec) values for each repetition were recorded.

For each modality independently, the following experiments were performed:

**Experiment 1**: To demonstrate the ability of the SWPCA algorithm to reduce undesired effects due to acquisition site, the PCA + SVC pipeline was applied to the datasets labelled by acquisition site. Classification accuracy was compared to datasets with and without SWPCA. VBM was then applied to identify the spatial location of the between‐site differences. This was undertaken on the whole database (ALL), and subgroups containing only ASD or CTL participants.

**Experiment 2**: The discrimination ability of each modality, acquired at different sites was assessed by classification performance of individuals from London (LON) and Cambridge (CAM) was separately assessed, using group (ASD and CTL) as the labels.

**Experiment 3**: To assess the impact of SWPCA on the datasets when characterizing the differences between ASD and CTL groups, the classification pipeline comprising PCA + SVC, as well as VBM and CBM, were applied to all participants with group as the labels.

## RESULTS

### Experiment 1: Effect of Acquisition Site

The first experiment was to demonstrate the ability of SWPCA to reduce the intensity variance related to acquisition site. To do so, we first performed a VBM analysis in all five modalities (qT1, qT2, synT1, GM and WM) separately, with the uncorrected (without applying SWPCA) and the corrected (after applying SWPCA) maps, using the acquisition site as labels.

**Figure 3:**
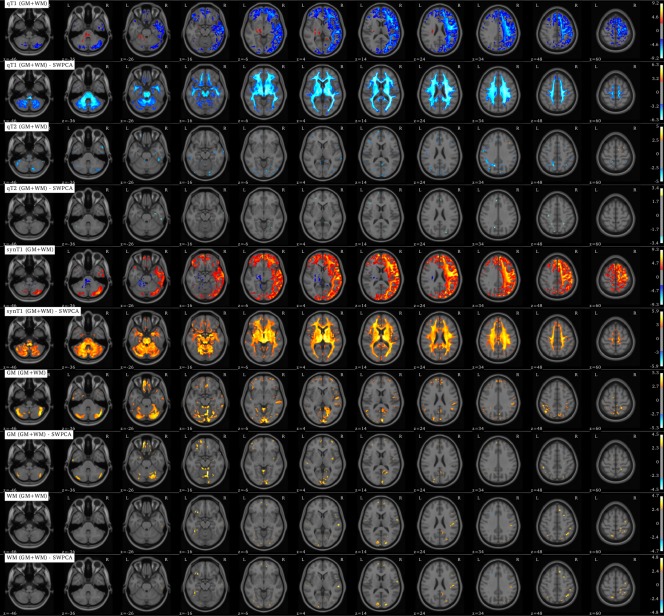
Brain *t*‐map (voxel‐based morphometry) of significant (*P* < 0.01, |*t*|>2.57) GM and WM between‐group differences using qT1, qT2, synT1, GM and WM modalities after applying SWPCA to remove site effects. [Color figure can be viewed at http://wileyonlinelibrary.com]

To illustrate where the sources of variance of the acquisition sites are located, Figure 3 shows a brain *t*‐map of significant (*P* < 0.01, |*t*|>2.57) GM and WM between‐site differences. The biggest reductions in variance were found in qT1 and synT1 maps, where high variability between acquisition sites, especially in the right hemisphere, was substantially reduced after the application of SWPCA. The reduction in the qT2, GM and WM maps was smaller, although noticeable.

To quantify the impact of this variance reduction on the between‐groups effects, the classification analysis was undertaken. Higher accuracy values imply that the maps contain site‐related patterns that were significant, whereas accuracy close to 0.5 indicates that the site‐related variance was low. The test was applied to ALL, and also to the ASD and CTL subgroups. The classification results are presented in Table 2.

**Table 2:** Between‐site classification accuracy ( ± standard deviation) for different modalities and masks without and with SWPCA correction

		ALL		CTL		ASD	
Modality	Mask	No SWPCA	SWPCA	No SWPCA	SWPCA	No SWPCA	SWPCA
qT1	GM+WM	0.875 ± 0.083	0.530 ± 0.130	0.847 ± 0.141	0.543 ± 0.115	0.769 ± 0.145	0.553 ± 0.093
	GM	0.849 ± 0.085	0.535 ± 0.107	0.835 ± 0.154	0.501 ± 0.090	0.712 ± 0.161	0.575 ± 0.084
	WM	0.865 ± 0.082	0.447 ± 0.071	0.876 ± 0.128	0.441 ± 0.058	0.813 ± 0.127	0.575 ± 0.153
qT2	GM+WM	0.596 ± 0.128	0.503 ± 0.093	0.615 ± 0.196	0.454 ± 0.075	0.506 ± 0.192	0.476 ± 0.103
	GM	0.596 ± 0.126	0.493 ± 0.097	0.549 ± 0.187	0.478 ± 0.108	0.497 ± 0.197	0.425 ± 0.091
	WM	0.612 ± 0.131	0.560 ± 0.128	0.576 ± 0.195	0.550 ± 0.146	0.541 ± 0.185	0.575 ± 0.172
synT1	GM+WM	0.904 ± 0.073	0.563 ± 0.060	0.919 ± 0.100	0.440 ± 0.057	0.807 ± 0.151	0.631 ± 0.098
	GM	0.879 ± 0.090	0.576 ± 0.035	0.899 ± 0.108	0.526 ± 0.079	0.800 ± 0.145	0.587 ± 0.042
	WM	0.904 ± 0.076	0.582 ± 0.047	0.894 ± 0.111	0.574 ± 0.038	0.859 ± 0.112	0.468 ± 0.101
GM	GM+WM	0.595 ± 0.133	0.586 ± 0.141	0.582 ± 0.192	0.566 ± 0.093	0.481 ± 0.169	0.468 ± 0.152
	GM	0.620 ± 0.141	0.585 ± 0.078	0.604 ± 0.227	0.574 ± 0.038	0.499 ± 0.188	0.525 ± 0.114
WM	GM+WM	0.659 ± 0.139	0.448 ± 0.066	0.635 ± 0.180	0.507 ± 0.144	0.522 ± 0.206	0.525 ± 0.198
	WM	0.639 ± 0.124	0.549 ± 0.072	0.578 ± 0.194	0.516 ± 0.126	0.549 ± 0.160	0.526 ± 0.136

Performance results indicate clear advantages of using SWPCA, in particular in the case of qT1 and synT1 which were associated with strong site‐dependent variance. These results are also consistent with the reduction of significant between‐group areas observed in Figure 3.

The between‐site differences were smaller for GM and WM maps, possibly due their reduced sensitivity. Since fractional occupancy values are abstract, unitless values derived from each image they are less influenced by the acquisition site effects. For qT2 maps, the site‐related differences were greater for the CTL participants than ASD where, according to the classification accuracy, they were nearly indistinguishable. Acquisition site differences were therefore noticeably reduced in the CTL and ALL databases, but not in the ASD.

### Experiment 2: Within‐Site between‐Group Differences

In this second experiment, accuracy, sensitivity and specificity in the between‐group comparison were recorded for images acquired from each site. This is an estimation of the discrimination ability of the different modalities without the influence of the site effects; Table 3. For all modalities, most of the values are close to a random classifier (∼50%), indicative of having either no significant differences between groups, or having spatially heterogeneous patterns of sMRI measures across individuals where mass‐univariate approaches are sub‐optimal in detecting group differences. It is interesting to note that the London sample contained more between‐group differences that those acquired in Cambridge.

**Table 3:** Classification accuracy (Acc), sensitivity (Sen) and specificity (Spec) ± standard deviation for each modality and mask using the participants acquired at the LON and CAM sites

		LONDON			CAMBRIDGE		
Modality	Mask	Acc	Sens	Spec	Acc	Sens	Spec
qT1	GM+WM	0.603 ± 0.175	0.512 ± 0.260	0.692 ± 0.237	0.504 ± 0.193	0.492 ± 0.276	0.515 ± 0.307
	GM	0.501 ± 0.157	0.440 ± 0.244	0.565 ± 0.245	0.484 ± 0.201	0.488 ± 0.300	0.480 ± 0.327
	WM	0.505 ± 0.174	0.485 ± 0.248	0.526 ± 0.242	0.451 ± 0.197	0.465 ± 0.297	0.435 ± 0.296
qT2	GM+WM	0.628 ± 0.168	0.535 ± 0.246	0.719 ± 0.237	0.467 ± 0.181	0.527 ± 0.307	0.417 ± 0.314
	GM	0.539 ± 0.149	0.425 ± 0.220	0.654 ± 0.222	0.491 ± 0.196	0.548 ± 0.316	0.430 ± 0.298
	WM	0.619 ± 0.194	0.585 ± 0.262	0.655 ± 0.250	0.472 ± 0.195	0.448 ± 0.283	0.492 ± 0.290
synT1	GM+WM	0.665 ± 0.158	0.578 ± 0.224	0.755 ± 0.238	0.479 ± 0.201	0.478 ± 0.318	0.475 ± 0.316
	GM	0.547 ± 0.159	0.475 ± 0.237	0.622 ± 0.252	0.514 ± 0.218	0.477 ± 0.322	0.555 ± 0.342
	WM	0.515 ± 0.185	0.520 ± 0.288	0.506 ± 0.254	0.509 ± 0.209	0.472 ± 0.317	0.542 ± 0.316
GM	GM+WM	0.513 ± 0.171	0.507 ± 0.252	0.518 ± 0.245	0.488 ± 0.202	0.445 ± 0.318	0.528 ± 0.285
	GM	0.586 ± 0.174	0.610 ± 0.247	0.564 ± 0.270	0.521 ± 0.187	0.522 ± 0.303	0.535 ± 0.289
WM	GM+WM	0.471 ± 0.181	0.455 ± 0.245	0.488 ± 0.278	0.489 ± 0.206	0.502 ± 0.319	0.483 ± 0.314
	WM	0.465 ± 0.174	0.445 ± 0.243	0.484 ± 0.268	0.468 ± 0.210	0.488 ± 0.292	0.448 ± 0.305

### Experiment 3: Effect of SWPCA on Group Differences

Finally, group differences were characterised with and without applying site‐effects reduction via SWPCA to the five modalities.

**Figure 4:**
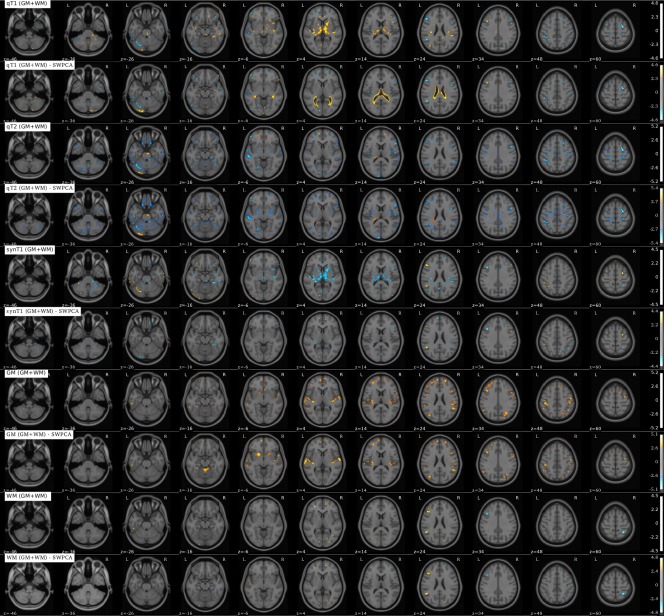
Brain *t*‐map (voxel‐based morphometry) of significant (*P* < 0.01, |*t*|>2.57) grey and white matter differences in ASD using qT1, qT2, synT1, GM and WM images before and after applying SWPCA to remove site effects. [Color figure can be viewed at http://wileyonlinelibrary.com]

Whole‐brain VBM analysis was performed on the corrected and uncorrected maps from each modality. Figure 4 depicts the brain *t*‐maps of significant (*P* < 0.01, |*t*|>2.57) qT1, qT2, synT1, GM and WM between‐group differences, using ALL, with the GM + WM mask, before and after applying SWPCA, so that the reduction of site‐related variability can be observed. Some of the highlighted areas after applying SWPCA are inconsistent across modalities, with spurious peaks and noise, including a large area around the ventricles in the qT1 and synT1 modalities related to some abnormal participants that will be discussed later. However, there were some areas that were consistent across modalities. Significant areas found across at least 4 of the 5 modalities correspond to the Advanced Automated Labelling (AAL) [Tzourio‐Mazoyer et al., 2002] areas of: (A) right superior frontal gyrus, Brodmann areas 6 (*z* = 60); (B) the pars opercularis of the left inferior frontal gyrus, Brodmann areas 44; (C) the pars triangularis of the left inferior frontal gyrus, Brodmann areas 45; (D) the posterior part of the left middle temporal gyrus (*z* = 24); CSF filled spaces on the margins of the ventricles (*z* = −6,4,14,24); and the left crus I of cerebellar hemisphere (*z* = −26).

**Figure 5:**
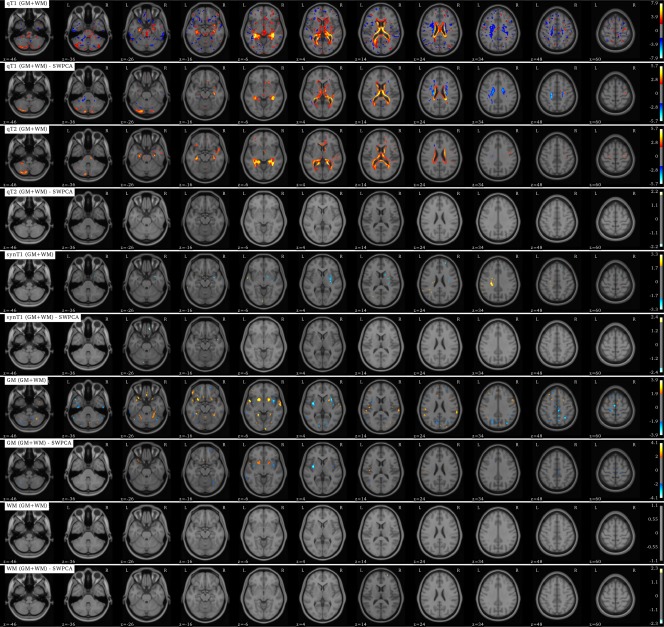
Brain *Z*‐map (component‐based morphometry) of significant (*P* < 0.01, |*Z*|>2.57) grey and white matter differences in ASD using qT1, qT2, synT1, GM and WM images before and after applying SWPCA to remove site effects. [Color figure can be viewed at http://wileyonlinelibrary.com]

The complementary CBM (Section 2.4) analysis was performed on the most significant components. The resulting regions, statistically thresholded with |*Z*|>2.57 (corresponding to *P* < 0.01), were superimposed on the MNI template, and are depicted in Figure 5. A reduction of significant between‐group areas after applying SWPCA is evident in most modalities, but particularly noticeable in the qT1 and qT2. In WM no significant regions were observed, neither before nor after SWPCA. The significant regions identified in any modality corresponded to the AAL areas of the CSF filled areas around the ventricles (planes *z* = −6, 4, 14, 24), the right middle temporal gyrus (plane *z* = 14) and the left crus I of cerebellar hemisphere (plane *z* = −26). However, none of these regions were repeated over more than two of the modalities, except for the large areas around ventricles that were caused by abnormalities in three participants, which will be discussed later.

Performance results for the classification analysis applied to ALL are shown in Table 4. Between‐group results were quite similar before or after applying SWPCA, although reducing between‐site variance generally reduced the performance towards a random classifier. The results in this table match the overall effects that were found in Figure 4, where most spurious significance peaks disappeared after applying SWPCA, but some regions were highlighted. These regions, where SWPCA did not seem to eliminate the significant areas but enhanced them, could be responsible for the accuracy increment in the analysis of the qT2 modality, and the GM with GM mask.

**Table 4:** Classification accuracy (Acc), sensitivity (Sen), and specificity (Spec)) ± standard deviation for the different modalities and masks using ALL, before and after applying SWPCA

		No SWPCA			SWPCA		
Modality	Mask	Acc	Sens	Spec	Acc	Sens	Spec
qT1	GM+WM	0.564 ± 0.123	0.503 ± 0.179	0.625 ± 0.177	0.435 ± 0.123	0.499 ± 0.181	0.371 ± 0.178
	GM	0.523 ± 0.112	0.468 ± 0.162	0.580 ± 0.192	0.458 ± 0.120	0.477 ± 0.187	0.441 ± 0.210
	WM	0.504 ± 0.131	0.475 ± 0.191	0.533 ± 0.194	0.484 ± 0.123	0.511 ± 0.179	0.456 ± 0.194
qT2	GM+WM	0.578 ± 0.115	0.487 ± 0.208	0.669 ± 0.178	0.593 ± 0.136	0.546 ± 0.206	0.640 ± 0.194
	GM	0.554 ± 0.135	0.492 ± 0.194	0.614 ± 0.181	0.526 ± 0.144	0.512 ± 0.209	0.543 ± 0.222
	WM	0.516 ± 0.138	0.508 ± 0.198	0.522 ± 0.216	0.499 ± 0.137	0.477 ± 0.209	0.521 ± 0.196
synT1	GM+WM	0.596 ± 0.132	0.509 ± 0.194	0.680 ± 0.172	0.577 ± 0.130	0.479 ± 0.208	0.676 ± 0.183
	GM	0.587 ± 0.139	0.509 ± 0.210	0.665 ± 0.169	0.483 ± 0.136	0.489 ± 0.218	0.480 ± 0.200
	WM	0.496 ± 0.139	0.500 ± 0.189	0.492 ± 0.194	0.487 ± 0.134	0.513 ± 0.189	0.461 ± 0.211
GM	GM+WM	0.498 ± 0.120	0.486 ± 0.197	0.507 ± 0.203	0.490 ± 0.123	0.514 ± 0.197	0.465 ± 0.182
	GM	0.574 ± 0.121	0.571 ± 0.189	0.579 ± 0.163	0.593 ± 0.127	0.602 ± 0.172	0.587 ± 0.190
WM	GM+WM	0.499 ± 0.132	0.506 ± 0.189	0.487 ± 0.181	0.521 ± 0.129	0.510 ± 0.209	0.532 ± 0.180
	WM	0.506 ± 0.143	0.488 ± 0.219	0.526 ± 0.197	0.507 ± 0.122	0.521 ± 0.165	0.492 ± 0.193

## DISCUSSION

Brain anatomical and functional differences between ASD participants and controls have been explored by a number of previous studies [Di Martino et al., 2014; Ecker et al., 2015; Hernandez et al., 2015; Lenroot and Yeung, 2013; Zürcher et al., 2015]. Many affected structures have been proposed in each of these studies, however as a recent large‐scale study points out [Haar et al., 2014], these are frequently inconsistent throughout the literature. Researchers argue that most of these structures are database‐dependent, and since many studies use multi‐site acquisition procedures, the variance introduced by each acquisition site is a probable source of Type I errors.

The technical and logistical drawbacks of multicentre studies are widely documented, including participant recruitment procedures [Pearlson, 2009] and technical effects that range from the usage of different equipment or acquisition parameters [Van Horn and Toga, 2009] to physical changes that affect the performance of MRI scanners across time [Pearlson, 2009]. There is general recognition that standardization is needed to ensure the uniformity of the acquired maps. Different approaches have been used in large‐scale studies, such as the Alzheimer's Disease Neuroimaging Initiative (ADNI) where human “phantoms” were used to perform a preparatory optimisation of MRI scanning platforms [Friedman and Glover, 2006].

There are two major types of site effects, regardless of their source: geometric distortions and intensity inhomogeneities. In this work, we focused on the latter, since much of the geometric distortion has been eliminated during acquisition (see section “Image Database”), and the DARTEL normalization and registration acts as a homogenizing step, reducing both between‐site and between‐subject geometric differences, substantially reducing the impact of the site‐related geometric differences.

Regarding intensity correction, in the MRC AIMS database used in this study [Ecker et al., 2012, 2013, 2015], a standardization procedure based on quantitative imaging [Deoni et al., 2008] was used to minimize inter‐site variance and improve the signal‐to‐noise contrast. However, as the between‐site analysis in “Experiment 1: Effect of Acquisition Site” section suggests, this strategy still results in variance that makes it easier to distinguish scanning sites than diagnostic groups. For example, when using qT1 the accuracy for LON vs. CAM classification was >80%, whilst when classifying ASD vs. CTL it was 52%. This marks the substantial effect of site variance on the maps’ intensity distribution, even when the multi‐site study employs quantitative imaging protocol on the same model of scanner platform across sites. However, with the inclusion of GM and WM maps, we can observe that the inhomogeneities found on qT1 or synT1 barely affected the segmentation procedure.

In this work, the approach we have taken is to perform a multivariate decomposition of each dataset into a number of components that explain different portions of variance. The following step was to identify the components of variance that are due to multi‐site acquisition and reduce them. Decomposition was completed using PCA and then, to identify which of the components were linked to acquisition site, we performed an ANOVA on the component scores. Finally, using the weighting function defined in Sec. 2.3.3, we reconstructed the original signal reducing the undesired variance, in what we called Significance Weighted PCA (SWPCA). The method has proven its ability in reducing undesired variance, quantifiable by means of the accuracy obtained in a site vs. site classification. In this case, SWPCA reduced the accuracy from >0.8 to approximately ∼0.5, a random classifier, suggesting that most site‐related variance was eliminated.

A simpler approach such as applying a voxel‐by‐voxel ANOVA would also be useful to reduce the acquisition site effects [Suckling et al., 2012]. However, SWPCA is a multivariate approach that still offers major advantages over this voxel‐wise algorithm, and similar algorithms have found utility in text document searches [Kriegel et al., 2008; Tavoli et al., 2013; Zhang and Nguyen, 2005]. First, PCA models the different sources of variance of the dataset, whereas a simple voxel‐wise ANOVA only removes mean site differences, which might result in less statistical power. Secondly, SWPCA is multivariate in nature, where each component contains information that potentially affects all voxels. Together, these two features allow SWPCA to identify the components linked to the undesired effects, and reduce their impact with a weighted reconstruction approach, reducing the general variance related to the acquisition site. However, this increased power reveals a major drawback: SWPCA needs at least a moderate number of participants to work properly. That is the reason why we cannot apply SWPCA to databases such as ADNI [Friedman and Glover, 2006] or ABIDE [Di Martino et al., 2014], where the number of participants acquired at each site is small, or to the six travelling phantoms used in the calibration of the MRC AIMS study.

There exist a number of similar multivariate methods that model the influence of categorical variables, such as the well‐known Partial Least Squares (PLS) algorithm [Vinzi et al., 2010] or Surrogate Variable Analysis (SVA) [Leek and Storey, 2007]. In the first case, both PLS and SWPCA take categorical variables *Y* along with the data *X* as inputs to partition the influence of these into components. However, the most significant difference is the underlying model. Whilst SWPCA estimates the principal components blindly using their variance, which is what we aim to reduce, and performs an ANOVA afterwards, PLS uses the categorical variable in the computation of the covariance matrix and then estimates the components.

On the other hand, SVA, used for gene expression studies [Leek and Storey, 2007], is more comparable to SWPCA. The SVA algorithm uses a number of decomposition and significance estimation steps to construct a set of surrogate variables; that is, variables that account for the unmodeled variance and expression heterogeneity. While similar to SWPCA in the steps used (i.e. SVD decomposition and significance estimation), their approaches are fundamentally different. SVA constructs a higher complexity model that starts by eliminating the contribution of primary variables to produce a number of unknown hidden (surrogate) variables, whereas SWPCA is intended to reduce complexity by producing variance‐reduced maps to reduce the influence of previously known, but unconsidered, variables and facilitate a subsequent analysis focused only on the relevant variables.

**Figure 6:**
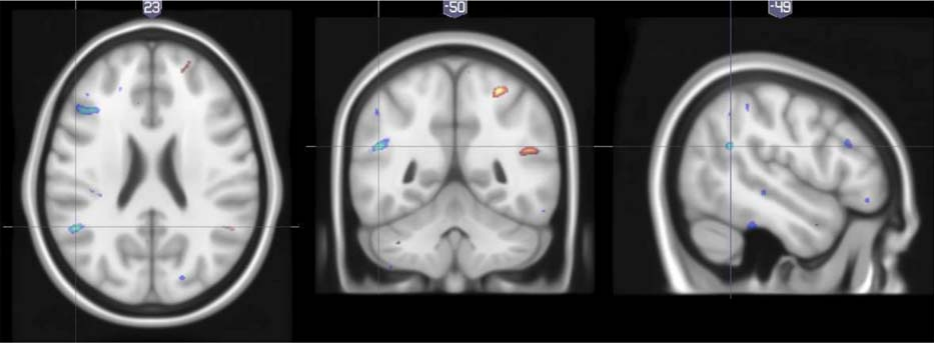
Location of the significant region that we have labelled D (posterior part of the superior temporal gyrus) within the MNI template.

Focusing on the VBM results, after performing the site‐effects removal by SWPCA significant between‐group differences were noted in five areas: (A) the right superior frontal gyrus; (B) the pars opercularis of the left inferior frontal gyrus; (C) the pars triangularis of the left inferior frontal gyrus; (D) the posterior part of the left middle temporal gyrus; and (E) the left crus I of cerebellar hemisphere. The first three regions are within Brodmann areas 6, 44 and 45. However, when examining the projection of the region D onto the MNI template (see Fig. 6), it is also located in the posterior part of the left superior temporal gyrus. Therefore, D corresponds closely with the region between Brodmann areas 22 and 39, the Temporo‐Parietal Junction (TPJ), with negative *t*‐value at the left side (containing Wernicke's area) and positive *t*‐value at the right side.

The role of these regions in autism has received much attention. Brodmann areas 44 and 45, that together make the Broca's Area (of importance in speech production and a proposed part of the human mirror neuron system (Nishitani et al., 2005)], is a region where mirror neuron dysfunction has been consistently reported in ASD‐affected children [Dapretto et al., 2006] and adults [Hadjikhani et al., 2006; Lopez‐Hurtado and Prieto, 2008; Verly et al., 2014]. Wernicke's area, contained in the left TPJ, is also linked to language, and has been associated with ASD in several works [Hadjikhani et al., 2006; Kriegel et al., 2008; Verly et al., 2014]. Additionally, the right TPJ has been proposed as related to mentalizing and has been repeatedly implicated in autism [Barnea‐Goraly et al., 2004], including a fMRI study of a subsample of this same AIMS dataset [Lombardo et al., 2011]. The right superior frontal gyrus (region A) is more equivocal, with some studies [Ecker et al., 2010, 2012] reporting abnormalities in this area, while others [Hadjikhani et al., 2006; Segovia et al., 2014] report no significant differences. Our analyses reveal no differences in the insula and amygdala, brain structures frequently linked to autism.

**Figure 7:**
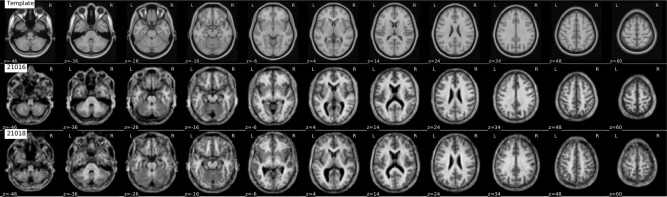
The template used in this work compared to two of the participants with abnormal ventricle size (21016 and 21018). Atrophy of the cerebellum in participant 21016 can also be appreciated, responsible for some of the ‘highlighted’ areas in qT1, qT2 and synT1 *t*‐maps (see **Fig. 4**).

Some regions, particularly in qT2, synT1 and segmented GM maps show potentially spurious significance peaks around the ventricles and especially in the left crus I of cerebellar hemisphere (region E). After examining the database, two individuals had appreciable structural abnormalities in the form of abnormal ventricle size and cerebellar atrophy, as can be seen in Figure 7. It is possible that these participants influenced the computation of the *t*‐maps, and therefore are responsible for the significance in region E and areas surrounding the ventricles and, since they are part of the LON subdataset, could also be responsible for the increased classification accuracy of the quantitative T1 and T2, and the synthetic T1 maps in this sub‐dataset.

After observing the influence of these participants on the computation of the *t*‐maps, we can assume that most of the structural differences in ASD are so subtle that the influence of just one or two images can impact on the final results. This, along with the poor performance of the classification pipeline presented in Section 3, dramatically reduces the significance of the aforementioned *t*‐maps. Therefore, the existing evidence leads to the conclusion that ASD presents as either undetectable structural differences or, more likely, with such heterogeneous differences that are difficult to establish a common pattern even after reducing the variance introduced by acquisition site.

It may be the case that cohorts of individuals examined at different sites are somehow systematically biased towards a specific type of patient (in ways that we cannot see simply based on phenotypic information), then site‐related intensity variability is also enriched with important variability about nested autism subgroups. So with any technique trying to remove the site‐related inhomogeneity, the subgroup information could also be removed. Together, the evidence supports the claim that defining meaningful subgroups based on different measures, such as genetic profiling, clinical co‐morbidities or sensory sensitivities, is the most urgent next step for ASD research [Haar et al., 2014].

## CONCLUSIONS

In this work, a novel method called Significance Weighted PCA (SWPCA) is proposed. The method comprises a principal component extraction, the characterization of their statistical significance according to a categorical variable (in this case, acquisition site) and the reconstruction of the images using a weighted approach. This approach was tested with an ASD database and demonstrated its ability for reducing inter‐site variance, which we have characterized with a site vs. site classification of the images. A priori, the images yielded >0.8 of accuracy, in contrast to a ∼0.5 accuracy between groups, suggesting that the images contained many differences depending on acquisition site. After minimizing site‐related variance, statistically significant group differences were found for example in Broca's area and the temporoparietal junction. However, their discriminative power was not sufficient to classify diagnostic groups, yielding accuracy results close to random. Our work supports recent claims that ASD is a highly heterogeneous syndrome/diagnostic category that is difficult to characterize globally using neuroimaging and therefore different (and more homogeneous) subgroups should be defined using imaging and perhaps other biological measures to obtain a deeper understanding of ASD.
